# Electric‐Field‐Controlled Interconversion of Antiferromagnetic States in a Two‐Dimensional Antiferroelectric Halide Perovskite

**DOI:** 10.1002/advs.77147

**Published:** 2026-08-14

**Authors:** Wan Zhao, Xiaodong Zhou, Tao Zhu, Jie Chen, Hang Li, Jingyan Chen, Meiling Xu, Wenhong Wang

**Affiliations:** ^1^ Institute of Quantum Materials and Devices School of Electronic and Information Engineering Tiangong University Tianjin China; ^2^ School of Physical Science and Technology Tiangong University Tianjin China; ^3^ Jiangsu Key Laboratory of Extreme Multi‐Field Materials Physics School of Physics and Electronic Engineering Jiangsu Normal University Xuzhou China

**Keywords:** altermagnetism, antiferroelectricity, multiferroicity, two‐dimensional halide perovskites, type‐IV magnetism

## Abstract

Electric‐field control of spin‐dependent electronic structures in antiferromagnets is highly desirable for low‐power spintronic devices, however, such control remains largely unexplored. Here, we propose a promising route based on electric‐field‐driven switching between symmetry‐distinct altermagnetic (AM) and type‐IV antiferromagnetic (AFM) states, enabling reversible interconversion between spin‐split and spin‐degenerate electronic structures. First‐principles calculations identify the organic‐inorganic halide perovskite multiferroic monolayer ${\left(C_{2}H_{5}{\mathrm{NH}}_{3}\right)}_{2}\left[{\mathrm{FeCl}}_{4}\right]$ as a candidate platform for realizing this concept. This monolayer possesses two inequivalent antiferroelectric (AFE) structures that host distinct AFM electronic states: one realizes an AM state with nonrelativistic spin splitting, whereas the other belongs to the type‐IV AFM class with spin‐degenerate bands in the nonrelativistic limit. Remarkably, an in‐plane electric field as low as 0.017 V/Å drives a transition between the two AFE phases, thereby switching the system between an AM spin‐split state and a spin‐degenerate type‐IV AFM state. Moreover, the two AFM states exhibit distinct, symmetry‐governed Kerr rotation responses, enabling optical detection of the electric‐field‐driven interconversion. These findings establish AFE interconversion as a ferroic‐order‐based route for electrically programming symmetry‐distinct AFM electronic states, providing a new route for low‐power, optically readable AFM spintronics.

## Graphical abstract

Electric‐field‐controlled antiferroelectric switching offers a ferroic pathway for reversible interconversion between symmetry‐distinct antiferromagnetic states in a two‐dimensional halide perovskite. A spin‐split altermagnet transforms into a spin‐degenerate type‐IV antiferromagnet through field‐driven structural symmetry reconstruction, enabling programmable spin‐dependent electronic structures and distinct Kerr optical fingerprints for electrically writable, optically readable antiferromagnetic spintronics.

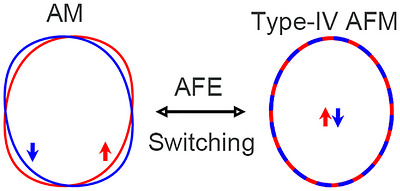

## 1 Introduction

Antiferromagnets offer key advantages for spintronic applications, including negligible stray fields, robustness against external magnetic perturbations, and ultrafast spin dynamics. Recent symmetry analyzes have shown that two‐dimensional (2D) collinear compensated antiferromagnets can be broadly classified into conventional antiferromagnets, altermagnets, and type‐IV antiferromagnets according to the symmetry operations connecting opposite‐spin sublattices [1, 2, 3, 4]. These subclasses exhibit fundamentally different spin‐dependent electronic structures, together with distinct transport and magneto‐optical responses. Realizing reversible switching between these spin‐dependent electronic structures within a single‐phase material would enable on‐demand control of antiferromagnetic (AFM) functionalities, including spin splitting, transport responses, and magneto‐optical readout.

Conventional magnetic‐field‐ or current‐based approaches mainly reorient the Néel vector and are effective for manipulating magnetic alignment, but they are not well suited for directly converting one symmetry‐distinct collinear AFM state into another. Moreover, their reliance on large fields or high current densities often leads to substantial energy consumption and Joule heating. To overcome these limitations, an appealing strategy is to use electric fields to drive ferroic structural phase transitions that reconstruct magnetic symmetry. Multiferroic systems provide a particularly suitable platform for this purpose, because electric polarization, lattice distortion, and magnetic order coexist and can be coupled through the underlying crystal structure [5, 6, 7]. In such systems, electric fields can transform inequivalent ferroic structural variants, such as different FE phases or different AFE phases [8, 9, 10]. If these structures possess distinct symmetry operations connecting opposite‐spin sublattices, such structural transitions can interconvert symmetry‐distinct AFM electronic states. Although FE‐ or AFE‐switchable magnetism has been widely explored [8, 9, 10, 11, 12, 13, 14, 15, 16, 17, 18, 19, 20, 21, 22, 23, 24], exploiting such ferroic structural transition to interconvert symmetry‐distinct collinear AFM states remains largely unexplored.

Here, we show that monolayer ${\left(C_{2}H_{5}{\mathrm{NH}}_{3}\right)}_{2}\left[{\mathrm{FeCl}}_{4}\right]$, a 2D organic‐inorganic halide perovskite multiferroic, provides an ideal platform for AFE‐structure‐mediated interconversion between symmetry‐distinct AFM electronic states. Using first‐principles calculations combined with symmetry analysis, we find that this monolayer hosts two inequivalent AFE structures that can be transformed under an in‐plane electric field. Importantly, these two AFE structures support fundamentally different compensated collinear AFM electronic states. One structure realizes an AM state with momentum‐dependent spin splitting, whereas the other belongs to the type‐IV AFM class, whose nonrelativistic bands remain spin degenerate. We further find that an electric field as low as 0.017 V/Å can drive the transition between these two AFE structures, thereby reconstructing the magnetic symmetry and enabling reversible interconversion between spin‐split and spin‐degenerate AFM electronic structures. The accompanying symmetry change also produces distinct off‐diagonal optical conductivity and Kerr rotation responses, providing an optical fingerprint for the electrically controlled symmetry‐distinct collinear AFM states.

## 2 Results

**FIGURE 1:**
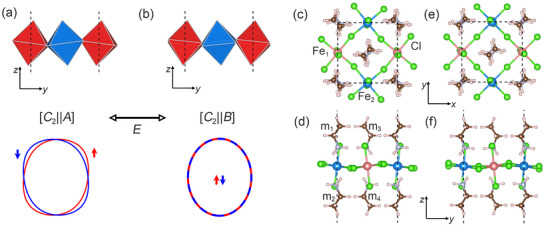
Conceptual illustration of electric‐field‐driven interconversion between AM and type‐IV AFM states and its material realization. (a) Schematic real‐space spin‐labeled octahedral configuration and momentum‐space alternating spin splitting of an AM state. The opposite‐spin sublattices are connected by $\left[C_{2}∥A\right]$, where A is a proper or improper real‐space rotation. Here, $A=M_{y}t$ or $C_{2y}t$ is shown as a representative example. (b) Schematic real‐space spin‐labeled octahedral configuration and momentum‐space electronic structure of a type‐IV antiferromagnetic state. In this case, the opposite‐spin sublattices are connected by $\left[C_{2}∥B\right]$, with $B\in \{M_{z}\left(t\right),C_{2z}\left(t\right)\}$, enforcing spin‐degenerate bands in the nonrelativistic limit. An in‐plane electric field E drives the interconversion between these two symmetry‐distinct collinear antiferromagnetic states. (c,d) Top and side views of the ${\mathrm{AFE}}_{1}$ monolayer derived from the bulk ${\left(C_{2}H_{5}{\mathrm{NH}}_{3}\right)}_{2}\left[{\mathrm{FeCl}}_{4}\right]$ Pcab phase. (e,f) Top and side views of the ${\mathrm{AFE}}_{2}$ monolayer derived from the bulk ${\left(C_{2}H_{5}{\mathrm{NH}}_{3}\right)}_{2}\left[{\mathrm{FeCl}}_{4}\right]$ Pccn phase. Each monolayer contains one Fe‐Cl inorganic sheet and four ethylammonium cations, labeled as $m_{1}$–$m_{4}$.

We first outline the symmetry basis for interconverting distinct compensated 2D collinear AFM states, as shown in Figure 1. In a collinear antiferromagnet, the electronic structure is governed by the symmetry operation that connects the opposite‐spin sublattices. Conventional compensated AFM states are characterized by opposite‐spin sublattices related by operations such as $\left[C_{2}∥P\right]$ or $\left[C_{2}∥t\right]$, where P and t denote spatial inversion and translation, respectively. These symmetries enforce spin‐degenerate bands in the nonrelativistic limit, and forbid Berry‐curvature‐driven anomalous transport and magneto‐optical responses. By contrast, altermagnets are characterized by $\left[C_{2}∥A\right]$, where A is a real‐space rotation or mirror operation that is neither translation nor inversion and does not belong to $M_{z}\left(t\right)$ or $C_{2z}\left(t\right)$. As schematically illustrated in Figure 1a, representative operations, such as $M_{y}t$ or $C_{2y}t$ connect opposite‐spin sublattices and generate momentum‐dependent alternating spin splitting. Type‐IV AFM states represent a different symmetry class, in which the opposite‐spin sublattices are connected by $\left[C_{2}∥B\right]$ with $B\in \{M_{z}\left(t\right),C_{2z}\left(t\right)\}$ [4]. Such operations enforce spin‐degenerate bands in the nonrelativistic limit, as shown in Figure 1b. Spin‐orbit coupling (SOC) can lift the symmetry‐enforced spin degeneracy, and generate anomalous transport and magneto‐optical responses. Thus, AM and type‐IV AFM states constitute two symmetry‐distinct compensated magnetic phases with different spin‐splitting patterns and functional responses. Their reversible interconversion would therefore enable on‐demand switching between spin‐split and spin‐degenerate AFM electronic structures. To realize such interconversion by an electric field, the key is to identify a structural degree of freedom that can be electrically switched and that simultaneously changes the symmetry operation connecting opposite‐spin sublattices.

Organic‐inorganic multiferroics are particularly appealing in this regard, because electric‐field‐responsive molecular dipoles from the organic cations are coupled to the magnetic inorganic framework. Reorientation of these molecular dipoles can distort magnetic inorganic framework, thereby reconstructing the lattice symmetry and the symmetry operations connecting opposite‐spin sublattices. The layered organic‐inorganic halide ${\left(C_{2}H_{5}{\mathrm{NH}}_{3}\right)}_{2}\left[{\mathrm{FeCl}}_{4}\right]$ is well suited for this purpose [25]. Its bulk structure consists of Fe‐Cl inorganic sheets separated by bilayers of ethylammonium cations. The Fe‐centered chloride octahedra form a corner‐sharing framework, while the organic cations interact with the apical Cl atoms through hydrogen bonding. The large interlayer separation of about 10.5–11 Å indicates weak interlayer coupling, suggesting that ${\left(C_{2}H_{5}{\mathrm{NH}}_{3}\right)}_{2}\left[{\mathrm{FeCl}}_{4}\right]$ monolayer can be exfoliated from the layered parent crystal. Moreover, the experimentally observed sequence of structural transitions in bulk ${\left(C_{2}H_{5}{\mathrm{NH}}_{3}\right)}_{2}\left[{\mathrm{FeCl}}_{4}\right]$ originates from coupled molecular reorientation and octahedral rotation/tilting, indicating strong coupling among molecular degrees of freedom, lattice distortion, and magnetic order.

Motivated by this structural flexibility, we construct two monolayer structures derived from the bulk Pcab and Pccn phases, denoted hereafter as ${\mathrm{AFE}}_{1}$ and ${\mathrm{AFE}}_{2}$, respectively, (Figures 1c–f). The two monolayers belong to the layer groups $p2_{1}/b11$ and p112/a, respectively. Each monolayer contains one Fe‐Cl inorganic sheet and four ethylammonium groups, labeled as $m_{1}$–$m_{4}$. The molecular pairs $m_{1}/m_{2}$ and $m_{3}/m_{4}$ are related by inversion and carry opposite local polar displacements, giving rise to an overall AFE arrangement. The transformation from ${\mathrm{AFE}}_{1}$ to ${\mathrm{AFE}}_{2}$ can be viewed as a collective molecular reorientation accompanied by a reconstruction of the inorganic framework. For example, rotating the $m_{1}$ and $m_{2}$ cations clockwise by $90^{\circ }$ converts the Pcab‐derived ${\mathrm{AFE}}_{1}$ structure into the Pccn‐derived ${\mathrm{AFE}}_{2}$ structure, while simultaneously modifying the rotation and tilting pattern of the Fe‐centered chloride octahedra. Energetically, ${\mathrm{AFE}}_{1}$ is lower than ${\mathrm{AFE}}_{2}$ by 0.06 eV/f.u. Both phases are dynamically stable, as confirmed by the absence of imaginary phonon frequencies throughout the Brillouin zone (see Figure S1).

Such organic‐cation‐mediated structural responses are consistent with those observed in related organic–inorganic hybrid perovskites, where electric‐field‐driven ferroic switching is closely associated with local molecular reorientation coupled to inorganic‐framework distortion. For example, polarization switching in ${\mathrm{DMAGeI}}_{3}$ can be described by stepwise rotation of DMA cations, whereas the field‐induced metaelectric response in TMCM‐${\mathrm{CdCl}}_{3}$ involves rotational ordering of ${\mathrm{TMCM}}^{+}$ cations, including both in‐plane and out‐of‐plane molecular rotations, together with distortion of the ${\mathrm{CdCl}}_{3}$ framework [26, 27]. These results establish ${\mathrm{AFE}}_{1}$ and ${\mathrm{AFE}}_{2}$ as realistic structural variants for investigating electric‐field‐driven interconversion between symmetry‐distinct compensated collinear AFM electronic states.

We next examine the magnetic and electronic structures of the two inequivalent AFE monolayers. For ${\mathrm{AFE}}_{1}$ (${\mathrm{AFE}}_{2}$), the AFM state is lower in energy than the ferromagnetic and nonmagnetic states by about 0.11 (0.12) and 4.07 (4.13) eV/f.u., respectively, establishing antiferromagnetism as the ground state in both structures. This magnetic ground‐state assignment remains robust against the choice of the effective Hubbard parameter U within a physically relevant range, confirming that the predicted AFM ordering is not an artifact of a specific U value (see Table S1). Since experimental realizations of 2D materials generally require a supporting substrate, lattice mismatch may introduce moderate strain into the monolayer. To examine the influence of such substrate‐induced strain, we further calculated the relative stability and magnetic energetics of ${\mathrm{AFE}}_{1}$ and ${\mathrm{AFE}}_{2}$ under biaxial strains from −4% to +4%. As shown in Figure S3, the energy difference $▵E=E_{AFE_{1}}-E_{AFE_{2}}$ remains negative throughout the entire strain range, indicating that ${\mathrm{AFE}}_{1}$ remains energetically favored over ${\mathrm{AFE}}_{2}$ without any strain‐induced reversal of their relative stability. In addition, the AFM configuration remains lower in energy than both the FM and nonmagnetic configurations for the two AFE phases at different strains (Table S2). These results demonstrate that both the relative stability of the AFE phases and the AFM ground state are robust against moderate substrate‐induced biaxial strain.

**FIGURE 2:**
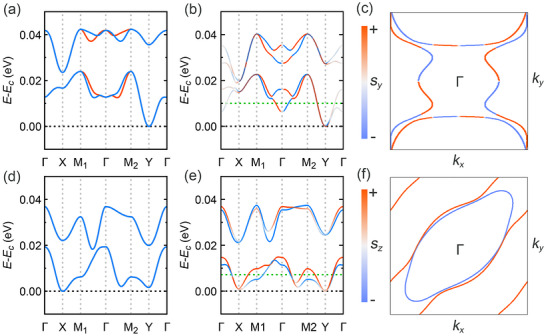
Symmetry‐distinct electronic structures of ${\mathrm{AFE}}_{1}$ and ${\mathrm{AFE}}_{2}$. (a–c) Electronic structure of ${\mathrm{AFE}}_{1}$: spin‐resolved band structure without SOC, spin‐resolved band structure with SOC, and spin‐resolved constant‐energy contour at the energy indicated by the green horizontal line in (b), respectively. (d–f) Electronic structure of ${\mathrm{AFE}}_{2}$: spin‐resolved band structure without SOC, spin‐resolved band structure with SOC, and spin‐resolved constant‐energy contour at the energy indicated by the green horizontal line in (e), respectively. Red and blue denote spin‐up and spin‐down components, respectively. For clarity, only the bands near the conduction band minimum are shown to highlight the spin‐splitting features; the complete band structures, including all occupied states, are provided in Figure S2.

Despite their similar magnetic energetics, the two AFE phases exhibit fundamentally different semiconducting electronic structures because they are governed by distinct spin symmetries (Figure 2). In the absence of SOC, ${\mathrm{AFE}}_{1}$ preserves the spin symmetries $\left[C_{2}∥M_{y}t\right]$ and $\left[C_{2}∥C_{2y}t\right]$, which impose $E\left(k_{x},k_{y},s\right)=E\left(k_{x},-k_{y},-s\right)$ and $E\left(k_{x},k_{y},s\right)=E\left(-k_{x},k_{y},-s\right)$, respectively. Together with Bloch periodicity, these relations enforce spin degeneracy along the $k_{x}=0,\pi$ and $k_{y}=0,\pi$ lines, while allowing spin splitting away from these high‐symmetry lines. Consistently, the spin‐resolved band structure of ${\mathrm{AFE}}_{1}$ displays momentum‐dependent alternating spin splitting (Figure 2a), identifying ${\mathrm{AFE}}_{1}$ as an altermagnet.

By contrast, ${\mathrm{AFE}}_{2}$ is governed by $\left[C_{2}∥M_{z}t\right]$ and $\left[C_{2}∥C_{2z}t\right]$, under which $E\left(k_{x},k_{y},s\right)=E\left(k_{x},k_{y},-s\right)$. The bands therefore remain spin degenerate throughout the Brillouin zone in the nonrelativistic limit (Figure 2d), identifying ${\mathrm{AFE}}_{2}$ as a type‐IV AFM. Upon inclusion of SOC, ${\mathrm{AFE}}_{1}$ largely retains its alternating spin‐splitting character along selected momentum paths, whereas ${\mathrm{AFE}}_{2}$ develops a SOC‐induced Zeeman‐like spin splitting [Figures 2b,e). The calculated spin‐resolved constant‐energy contours [Figures 2c,f) further highlight this contrast: ${\mathrm{AFE}}_{1}$ exhibits an alternating spin‐polarization pattern associated with AM symmetry, whereas ${\mathrm{AFE}}_{2}$ displays a SOC‐induced Zeeman‐like spin splitting characteristic of a type‐IV AFM state. Thus, although the two AFE phases share semiconducting AFM ground states, they host sharply distinct symmetry‐protected spin‐splitting patterns. Since the parent bulk structure contains a two‐layer stacking motif, we also examined bulk‐stacked bilayers exfoliated from the corresponding bulk phases. As discussed in the Figure S4, these bulk‐derived bilayers preserve the similar symmetry‐distinct magnetic physics: the ${\mathrm{AFE}}_{1}$ bilayer retains the altermagnetic state, whereas the ${\mathrm{AFE}}_{2}$ bilayer retains the spin‐degenerate type‐IV AFM state.

**FIGURE 3:**
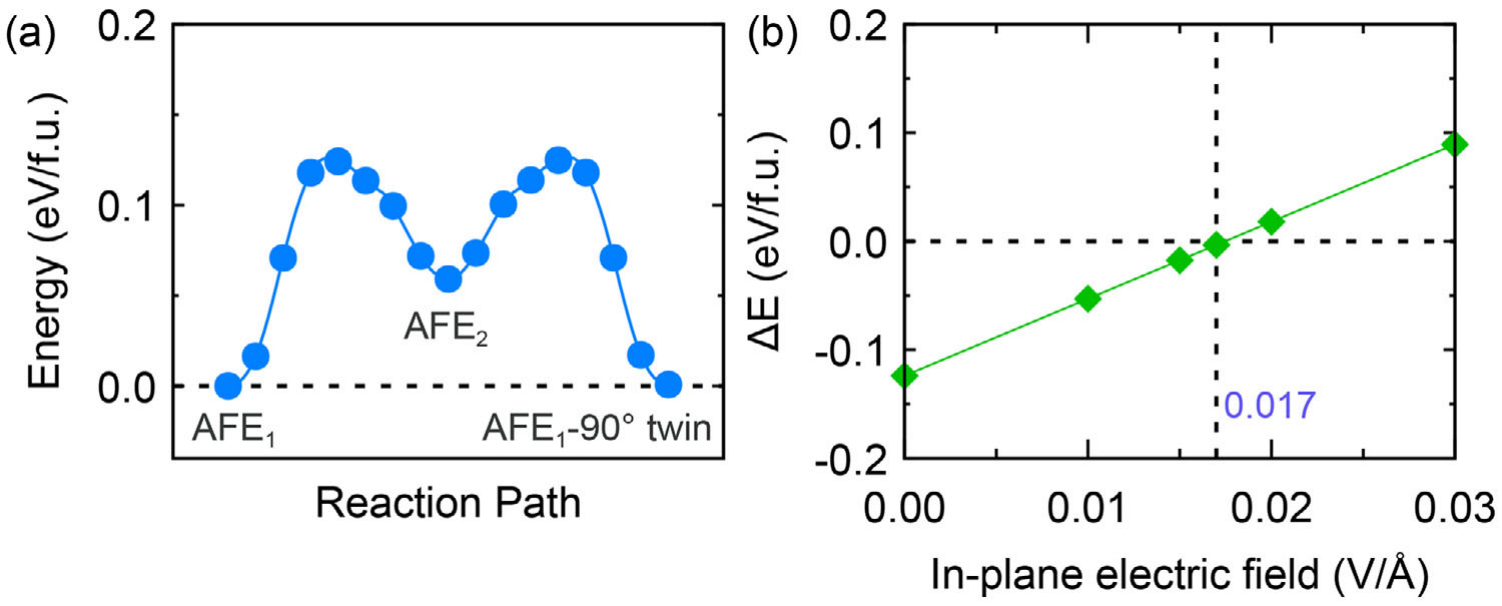
Electric‐field‐driven interconversion between ${\mathrm{AFE}}_{1}$ and ${\mathrm{AFE}}_{2}$ structures. (a) Energy profile along the switching pathway from ${\mathrm{AFE}}_{1}$ to its $90^{\circ }$ twin. Along this pathway, the $m_{1}$ and $m_{2}$ organic cations first rotate clockwise by $90^{\circ }$, converting ${\mathrm{AFE}}_{1}$ into ${\mathrm{AFE}}_{2}$; a subsequent clockwise $90^{\circ }$ rotation of the $m_{3}$ and $m_{4}$ cations transforms ${\mathrm{AFE}}_{2}$ into the ${\mathrm{AFE}}_{1}$‐$90^{\circ }$ twin. (b) Variation of the transition barrier from the ${\mathrm{AFE}}_{1}$ to ${\mathrm{AFE}}_{2}$ as a function of the applied in‐plane electric field.

The electric‐field‐driven phase transition between the two AFE phases is further examined. As shown in Figure 3a, the transformation from ${\mathrm{AFE}}_{1}$ to its $90^{\circ }$ twin proceeds through a metastable ${\mathrm{AFE}}_{2}$ configuration. The associated switching barrier is ∼0.12 eV/f.u. This moderate barrier is comparable to the calculated stepwise switching barriers in the hybrid halide perovskite ${\mathrm{DMAGeI}}_{3}$ [26], indicates that the molecular‐reorientation‐mediated structural reconstruction is energetically accessible.

Because the structural transformation is dominated by clockwise rotation of the organic cations about the z axis, the conjugate driving field is expected to lie in the plane. Indeed, when an external electric field is applied along the +x direction to ${\mathrm{AFE}}_{1}$, the system is driven across the barrier and transforms into ${\mathrm{AFE}}_{2}$ once the field strength reaches 0.017V/Å (see Figure 3b). This field strength is within the range attainable in gated 2D material devices [28, 29]. After the electric field is removed, the system remains in the ${\mathrm{AFE}}_{2}$ local minimum on the zero‐field energy landscape, suggesting a remanent, metastable structural switching behavior rather than a purely field‐induced transient state. Furthermore, starting from ${\mathrm{AFE}}_{2}$, a further clockwise rotation of the $m_{3}$ and $m_{4}$ organic groups drives the system into another ${\mathrm{AFE}}_{1}$ state (${\mathrm{AFE}}_{1}$‐$90^{\circ }$‐twin). The corresponding barrier profile is symmetric to that of the ${\mathrm{AFE}}_{1}\to$ ${\mathrm{AFE}}_{2}$ process.

It should be noted that AFE switching is generally more challenging than conventional FE polarization reversal. In an AFE structure, the antipolar arrangement leads to zero net polarization and multiple nearly degenerate domain variants, making deterministic selection of a target state less straightforward. Nevertheless, recent experiments have demonstrated that antipolar variants in AFE membranes can be selectively and reversibly manipulated by tailoring built‐in electric fields, enabling nonvolatile switching between distinct in‐plane and out‐of‐plane AFE domain configurations [30]. These results suggest that, although AFE switching is intrinsically more complex than FE switching, deterministic control of antipolar states is physically achievable when an appropriate field‐coupled structural pathway is available.

The symmetry‐distinct electronic structures of ${\mathrm{AFE}}_{1}$ and ${\mathrm{AFE}}_{2}$ naturally lead to distinct optical responses, providing experimentally accessible fingerprints for distinguishing the two AFE magnetic states. In principle, both electrical and optical probes can be used to detect their underlying electronic structures. Because the present system is semiconducting, optical probes are particularly attractive for imaging symmetry‐dependent electronic properties. Among them, the magneto‐optical Kerr effect (MOKE) has emerged as a powerful tool for characterizing magnetic order in 2D systems, as demonstrated in ${\mathrm{CrI}}_{3}$ [31], ${\mathrm{Cr}}_{2}{\mathrm{Ge}}_{2}{\mathrm{Te}}_{6}$ [32], and, more recently, AM ${\mathrm{Fe}}_{2}O_{3}$ [33, 34]. The Kerr response is governed primarily by the off‐diagonal optical conductivity $\sigma_{xy}\left(\omega \right)$, whose presence or absence can often be determined directly from magnetic symmetry.

**FIGURE 4:**
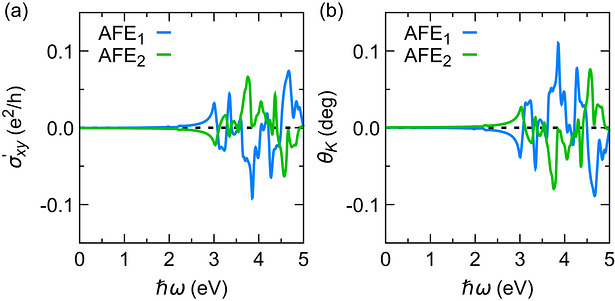
Optical fingerprint of ${\mathrm{AFE}}_{1}$ and ${\mathrm{AFE}}_{2}$. (a) Real parts of the off‐diagonal optical conductivity $\sigma_{xy}\left(\omega \right)$ and (b) the corresponding Kerr rotation angles for ${\mathrm{AFE}}_{1}$ and ${\mathrm{AFE}}_{2}$. The results are shown for representative Néel‐vector orientations, with the Néel vector along the y direction for ${\mathrm{AFE}}_{1}$ and along the x direction for ${\mathrm{AFE}}_{2}$. The symmetry‐governed magneto‐optical responses are consistent with the analysis summarized in Table 1.

For ${\mathrm{AFE}}_{1}$, when the Néel vector is aligned along the z direction, the system belongs to the magnetic space group $P2_{1}/c=\left\{E,P,M_{y}t,C_{2y}t\right\}$. Under both $M_{y}t$ and $C_{2y}t$, $\sigma_{xy}\left(\omega \right)$ changes sign and is therefore forced to vanish. As a result, ${\mathrm{AFE}}_{1}$ does not exhibit a Kerr response in this configuration. When the Néel vector rotates into the plane, however, $\sigma_{xy}\left(\omega \right)$ becomes strongly angle dependent and can periodically appear or disappear according to the corresponding magnetic symmetry. As summarized in Table 1, when the Néel vector lies along the ±x directions, the magnetic space group remains $P2_{1}/c$, so that $\sigma_{xy}\left(\omega \right)$ is still symmetry forbidden. Once the Néel vector deviates from the ±x axis, $\sigma_{xy}\left(\omega \right)$ generally becomes allowed. A particularly important case occurs when the Néel vector points along the ±y directions, for which the magnetic space group becomes $P2_{1}^{\prime }/c^{\prime }=\left\{E,P,TM_{y}t,TC_{2y}t\right\}$. In this case, the remaining symmetries no longer prohibit $\sigma_{xy}\left(\omega \right)$, and our first‐principles calculations yield a finite off‐diagonal optical conductivity (Figure 4a), giving rise to a nonzero Kerr rotation angle (Figure 4b).

**TABLE 1:** Magnetic space groups and the existence of off‐diagonal optical conductivity elements for ${\mathrm{AFE}}_{1}$ and ${\mathrm{AFE}}_{2}$ as a function of the in‐plane Néel‐vector angle φ. Here φ is measured from the x axis.

System / φ	$0^{\circ }$	$30^{\circ }$	$60^{\circ }$	$90^{\circ }$	$120^{\circ }$	$150^{\circ }$	$180^{\circ }$
${\mathrm{AFE}}_{1}$ / MSG($\sigma_{xy}$)	$P2_{1}/c$ (×)	P−1(✓)	P−1(✓)	$P2_{1}^{\prime }/c^{\prime }$(✓)	P−1(✓)	P−1(✓)	$P2_{1}/c$ (×)
${\mathrm{AFE}}_{2}$ / MSG($\sigma_{xy}$)	P2/c(✓)	P2/c(✓)	P2/c(✓)	P2/c(✓)	P2/c(✓)	P2/c(✓)	P2/c(✓)

${\mathrm{AFE}}_{2}$ exhibits a qualitatively different symmetry response. When the Néel vector is oriented along z, the magnetic space group is $P2^{\prime }/c^{\prime }=\left\{E,P,TM_{z}t,TC_{2z}t\right\}$. Similar to the out‐of‐plane configuration of ${\mathrm{AFE}}_{1}$, $\sigma_{xy}\left(\omega \right)$ is odd under these symmetry operations and is therefore forbidden. Once the Néel vector lies in plane, however, the situation differs sharply from ${\mathrm{AFE}}_{1}$. For any in‐plane orientation of the Néel vector, the magnetic space group remains $P2/c=\{E,P,M_{z}t,C_{2z}t\}$, under which $\sigma_{xy}\left(\omega \right)$ is symmetry allowed. Consequently, ${\mathrm{AFE}}_{2}$ supports a finite off‐diagonal optical conductivity and a nonzero Kerr response throughout the in‐plane rotation of the Néel vector. As shown in Figure 4, both $\sigma_{xy}\left(\omega \right)$ and the Kerr rotation angle are finite when the Néel vector is aligned along the x direction. This behavior contrasts sharply with ${\mathrm{AFE}}_{1}$, which remains magneto‐optically silent for the same Néel‐vector orientation. Such symmetry‐selective optical contrast provides a direct fingerprint for distinguishing the two electrically interconvertible antipolar AFM states.

Quantitatively, the calculated Kerr rotation angle reaches approximately 0.1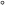, which is highly competitive among compensated magnetic systems. This value is larger than that reported for noncollinear antiferromagnetic ${\mathrm{Mn}}_{3}Y$ (Y = Sn, Ge, Ga; ∼0.02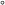) [35], and is comparable to those of altermagnetic ${\mathrm{Fe}}_{2}O_{3}$ (∼0.1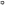) [33], the 2D compensated ferrimagnetic ${\mathrm{CrI}}_{3}$/${\mathrm{In}}_{2}{\mathrm{Se}}_{3}$/${\mathrm{CrI}}_{3}$ heterostructure (∼0.12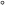) [36], and ${\mathrm{Nb}}_{3}I_{8}$ (∼0.13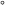) [37]. It is also of the same order of magnitude as the larger Kerr rotation angles reported for noncollinear antiferromagnetic ${\mathrm{Mn}}_{3}X$ (X = Ir, Rh, Pt; ∼ 0.6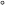) [38], ${\mathrm{Mn}}_{3}ZN$ (Z = Ga, Zn, Ag, Ni; ∼0.4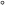) [39], and altermagnetic ${\mathrm{RuO}}_{2}$ (∼0.62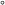) [40]. These comparisons indicate that the predicted MOKE signal should be readily detectable and can serve as a practical optical fingerprint for the electrically switched AFM states.

The present AFE switching mechanism should be viewed as a representative realization of a broader design principle rather than an isolated case. The essential requirement is not antiferroelectricity itself, but an electric‐field‐driven structural transformation capable of modifying the symmetry operation that connects opposite‐spin sublattices. Similar interconversion between symmetry‐distinct AFM states may therefore be realized in FE–FE or FE–AFE transitions, provided that the accompanying polar distortions, molecular reorientations, or octahedral reconstructions alter the sublattice‐connecting symmetry. This generality extends the significance of the present work beyond the specific material studied here and establishes ferroic structural switching as a versatile strategy for electrically programming AFM electronic structures and their magneto‐optical responses in 2D systems.

## 3 Conclusion

In conclusion, we have proposed an electric‐field‐driven route for interconverting symmetry‐distinct compensated collinear AFM states in 2D multiferroics. Using monolayer ${\left(C_{2}H_{5}{\mathrm{NH}}_{3}\right)}_{2}\left[{\mathrm{FeCl}}_{4}\right]$ as a prototype, we show that two inequivalent AFE structures, ${\mathrm{AFE}}_{1}$ and ${\mathrm{AFE}}_{2}$, host fundamentally different AFM electronic states. ${\mathrm{AFE}}_{1}$ realizes an AM state with nonrelativistic momentum‐dependent spin splitting, whereas ${\mathrm{AFE}}_{2}$ belongs to the type‐IV AFM class, whose bands remain spin degenerate in the nonrelativistic limit. This distinction originates from the different symmetry operations connecting the opposite‐spin sublattices. We further show that ${\mathrm{AFE}}_{1}$ and ${\mathrm{AFE}}_{2}$ can be interconverted through collective molecular reorientation and ${\mathrm{FeCl}}_{6}$ octahedral reconstruction. Importantly, an in‐plane electric field of 0.017V/Å drives the ${\mathrm{AFE}}_{1}$‐to‐${\mathrm{AFE}}_{2}$ transition, enabling switching between spin‐split and spin‐degenerate AFM electronic structures. This process also leads to distinct magneto‐optical responses, with the off‐diagonal optical conductivity and Kerr rotation serving as optical fingerprints of the electrically controlled AFM states. These results establish AFE structure phase transition as an effective ferroic‐order‐mediated route for engineering electrically writable and optically readable AFM functionalities in 2D materials.

## 4 Experimental Section

Electronic‐structure calculations were carried out within the framework of density functional theory using the projector augmented‐wave (PAW) method [41], as implemented in the Vienna ab initio Simulation Package (vasp) [42]. Exchange‐correlation effects were described by the generalized gradient approximation in the Perdew‐Burke‐Ernzerhof (PBE) form [43]. To capture the correlation effect of Fe 3d electrons, the GGA+U scheme [44] was adopted with an effective Hubbard parameter of U=2 eV. The different U of 2‐5 eV was checked, as summarized in Figure S2 and Table S1. During structural optimization, all atomic positions were fully relaxed until the residual force on each atom was below 0.001 eV/Å. A plane‐wave cutoff energy of 500 eV, an electronic convergence criterion of 10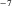 eV, and an 11×11×1 Monkhorst–Pack k‐point mesh were employed for the self‐consistent calculations. A vacuum thickness of at least 15 Å is used to avoid interaction between adjacent layers. The weak interlayer interaction was treated using the DFT‐D3 van der Waals correction [45, 46]. Phonon dispersions were obtained within density functional perturbation theory (DFPT) [47]. Maximally localized Wannier functions (MLWFs) were constructed using the wannier90 code [48] from a uniform 8×8×1 k‐mesh, including 48 Cl‐p orbitals and 20 Fe‐d orbitals. The energy barriers and switching pathways were determined by the climbing‐image nudged elastic band (CI‐NEB) method [49, 50]. An in‐plane electric field was introduced by applying a finite homogeneous field using the discretized perturbation method [51, 52].

The optical Hall conductivity [48] was calculated as

(1) $$\sigma_{xy}\left(\omega \right)=\frac{ie^{2}\hbar }{N_{k}V_{c}}\sum_{k}\sum_{n,m}\frac{f_{mk}-f_{nk}}{E_{mk}-E_{nk}}\frac{\langle \psi_{nk}|{\hat{v}}_{x}|\psi_{mk}\rangle \langle \psi_{mk}|{\hat{v}}_{y}\left|\psi_{nk}\right\rangle }{E_{mk}-E_{nk}-\left(\hbar \omega +i\eta \right)},$$

using a dense 501×501×1 k‐point mesh. Here, $V_{c}$ denotes the cell volume, $N_{k}$ is the total number of sampled k points, ℏω is the photon energy, and η represents the smearing factor.

For the 2D system, the magneto‐optical Kerr rotation angle was evaluated according to [53, 54, 55, 56]

(2) θK=Rei2ωdcσxyσxxs,

where $\theta_{K}$ is the Kerr rotation angle, c is the speed of light in vacuum, d is the effective thickness of the 2D material (10.844 Å for ${\mathrm{AFE}}_{1}$, 10.838 Å for ${\mathrm{AFE}}_{2}$), $\sigma_{xy}$ is the off‐diagonal optical conductivity of the magnetic layer, and $\sigma_{xx}^{s}$ is the diagonal optical conductivity of the nonmagnetic substrate, for which ${\mathrm{SiO}}_{2}$ was adopted in the present calculations.

## Conflicts of Interest

The authors declares no conflicts of interest.

## Supporting information


**Supporting File**: advs77147‐sup‐0001‐SuppMat.pdf.

## Data Availability

The data that support the findings of this study are available from the corresponding author upon reasonable request.
