# Supplementary material for: Electric‐Field‐Controlled Interconversion of Antiferromagnetic States in a Two‐Dimensional Antiferroelectric Halide Perovskite
Source: Adv Sci (Weinh). 2026 Aug 14:e77147. Online ahead of print. doi: 10.1002/advs.77147 (PMC13474192; doi:10.1002/advs.77147)
Supplement: Supplementary file 1 [file ADVS-9999-e77147-s001.pdf]

Supporting information for "Electric-Field-Controlled Interconversion of Antiferromagnetic States in a Two-Dimensional Antiferroelectric Halide Perovskite"

Wan Zhao Xiaodong Zhou Tao Zhu Jie Chen Hang Li Jingyan Chen Meiling Xu* Wenhong Wang**

Wan Zhao, Xiaodong Zhou, Tao Zhu, Jie Chen, Hang Li, Wenhong Wang
Institute of Quantum Materials and Devices, School of Electronic and Information Engineering, Tiangong University, Tianjin 300387, China
School of Physical Science and Technology, Tiangong University, Tianjin 300387, China
Email Address: zhouxiaodong@tiangong.edu.cn, wenhongwang@tiangong.edu.cn

Jingyan Chen, Meiling Xu
Jiangsu Key Laboratory of Extreme Multi-Field Materials Physics, School of Physics and Electronic Engineering, Jiangsu Normal University, Xuzhou 221116, China
Email Address: xml@calypso.cn

Table S1: Relative energies of AFM, FM, and non-magnetic states for AFE_1 and AFE_2 calculated with different Hubbard U values. For each antiferroelectric state and each U value, the AFM state is taken as the energy reference. Although the parent bulk *Pccn* phase is experimentally observed above the Néel temperature, our calculations focus on its isolated monolayer counterpart, where the AFM configuration is energetically favored and dynamically stable. This justifies using the *Pccn*-derived AFE_2 monolayer as a symmetry-distinct magnetic state for exploring electric-field-driven interconversion.

Structure	U (eV)	AFM (eV/f.u.)	FM (eV/f.u.)	Non-mag (eV/f.u.)
AFE_1	2	0	0.11	4.07
AFE_1	3	0	0.08	4.41
AFE_1	4	0	0.06	4.74
AFE_1	5	0	0.05	5.05
AFE_2	2	0	0.12	4.13
AFE_2	3	0	0.09	4.46
AFE_2	4	0	0.07	4.77
AFE_2	5	0	0.05	5.08

Table S2: Relative energies of AFM, FM, and non-magnetic states for AFE_1 and AFE_2 calculated under biaxial strain. For each state, the AFM state is taken as the energy reference.

Structure	Strain	AFM (eV/f.u.)	FM (eV/f.u.)	Non-mag (eV/f.u.)
AFE_1	-0.04	0	0.10	3.73
AFE_1	0.00	0	0.11	4.07
AFE_1	0.04	0	0.10	4.49
AFE_2	-0.04	0	0.14	3.75
AFE_2	0.00	0	0.12	4.13
AFE_2	0.04	0	0.10	4.54

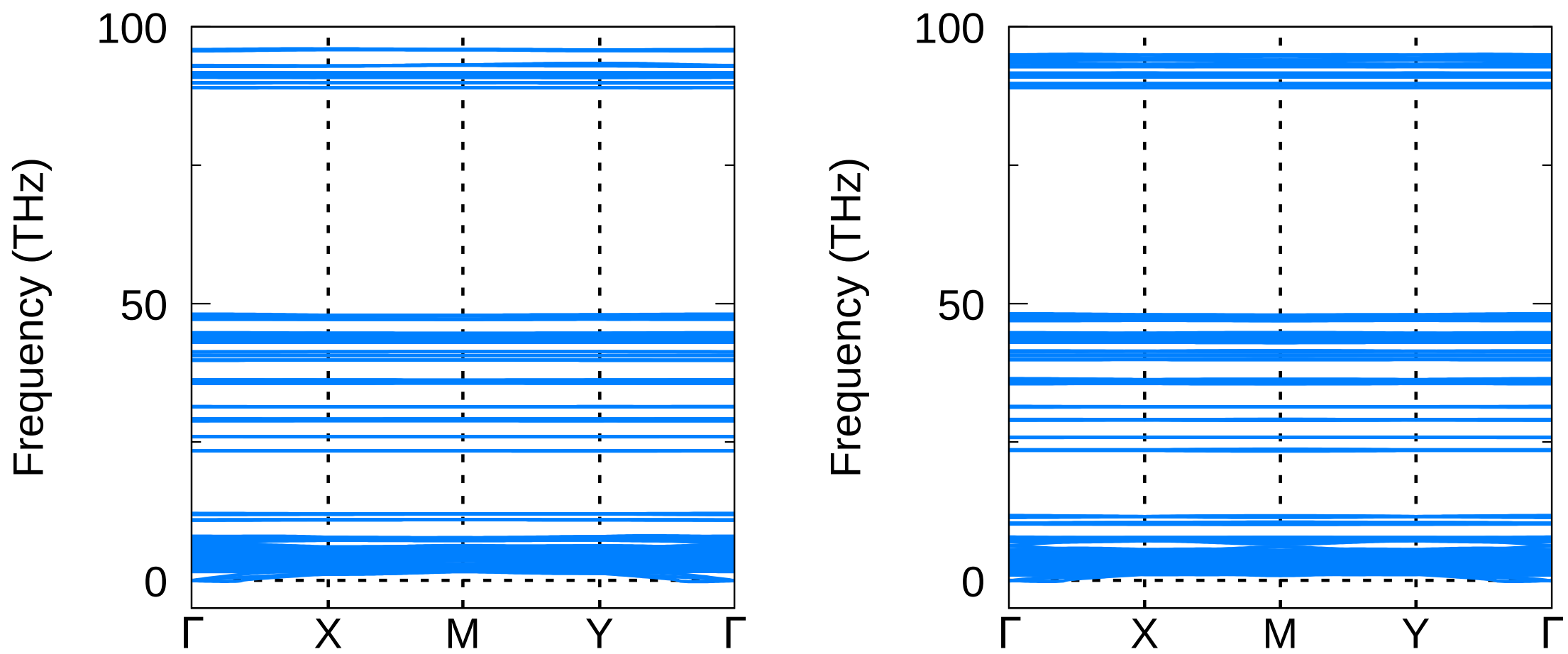

Figure S1: Phonon spectra of (a) AFE_1 and (b) AFE_2. Both antiferroelectric states exhibit almost no imaginary phonon frequencies throughout the Brillouin zone, indicating their dynamical stability.

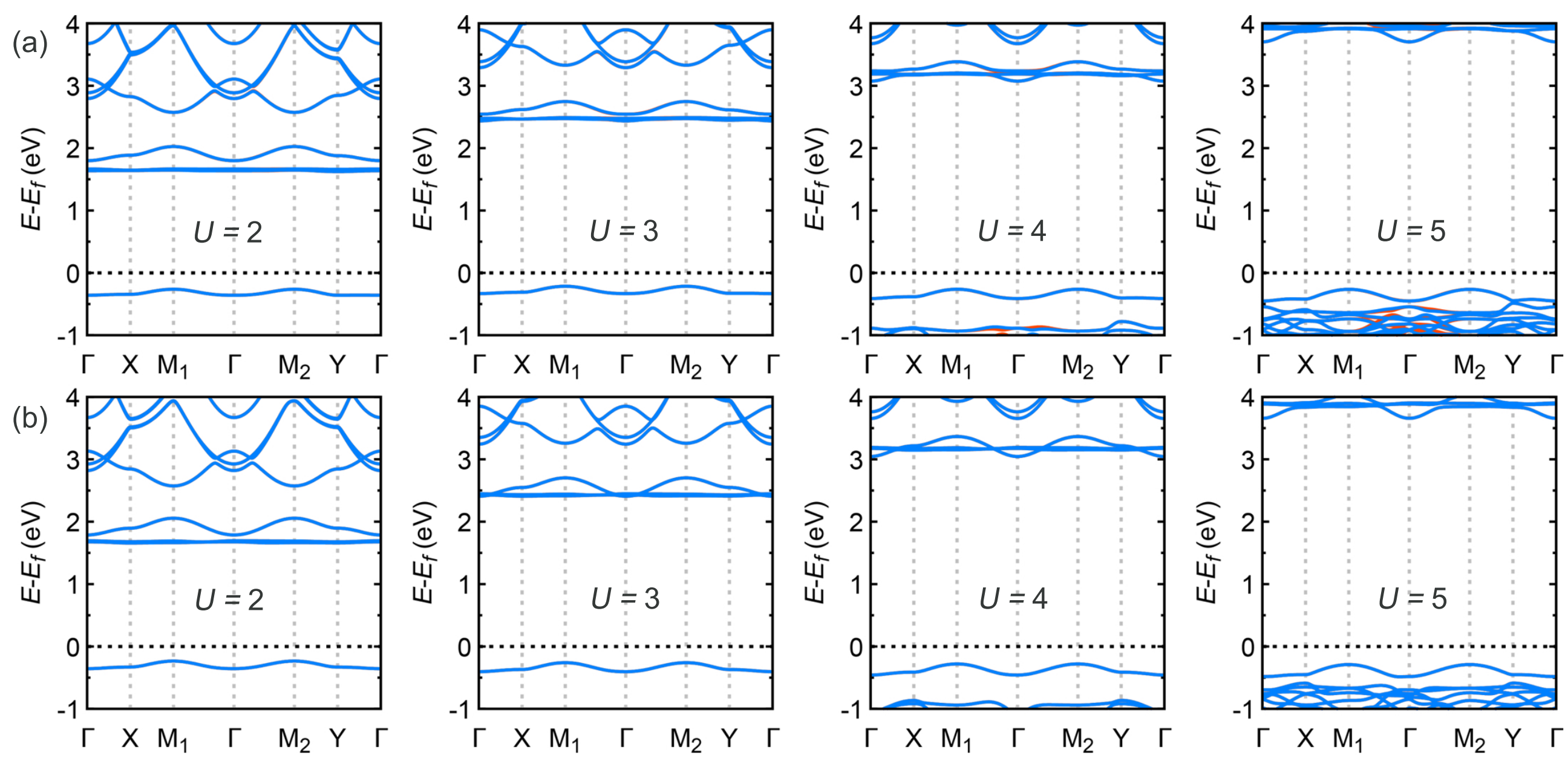

Figure S2: Spin-polarized band structures of (a) AFE_1 and (b) AFE_2 calculated with different Hubbard U values of 2, 3, 4, and 5 eV. With increasing U, both antiferroelectric states remain semiconducting, and their band gaps are gradually enlarged. The essential magnetic features are preserved over the considered U range: AFE_1 exhibits altermagnetism, whereas AFE_2 corresponds to a type-IV antiferromagnetic state. It should be noted that the band structures are plotted over a relatively wide energy range; therefore, the spin-splitting feature of AFE_1 is not visually prominent in this figure. A clearer representation of the spin splitting in AFE_1 is provided in Fig. 2 in the main text.

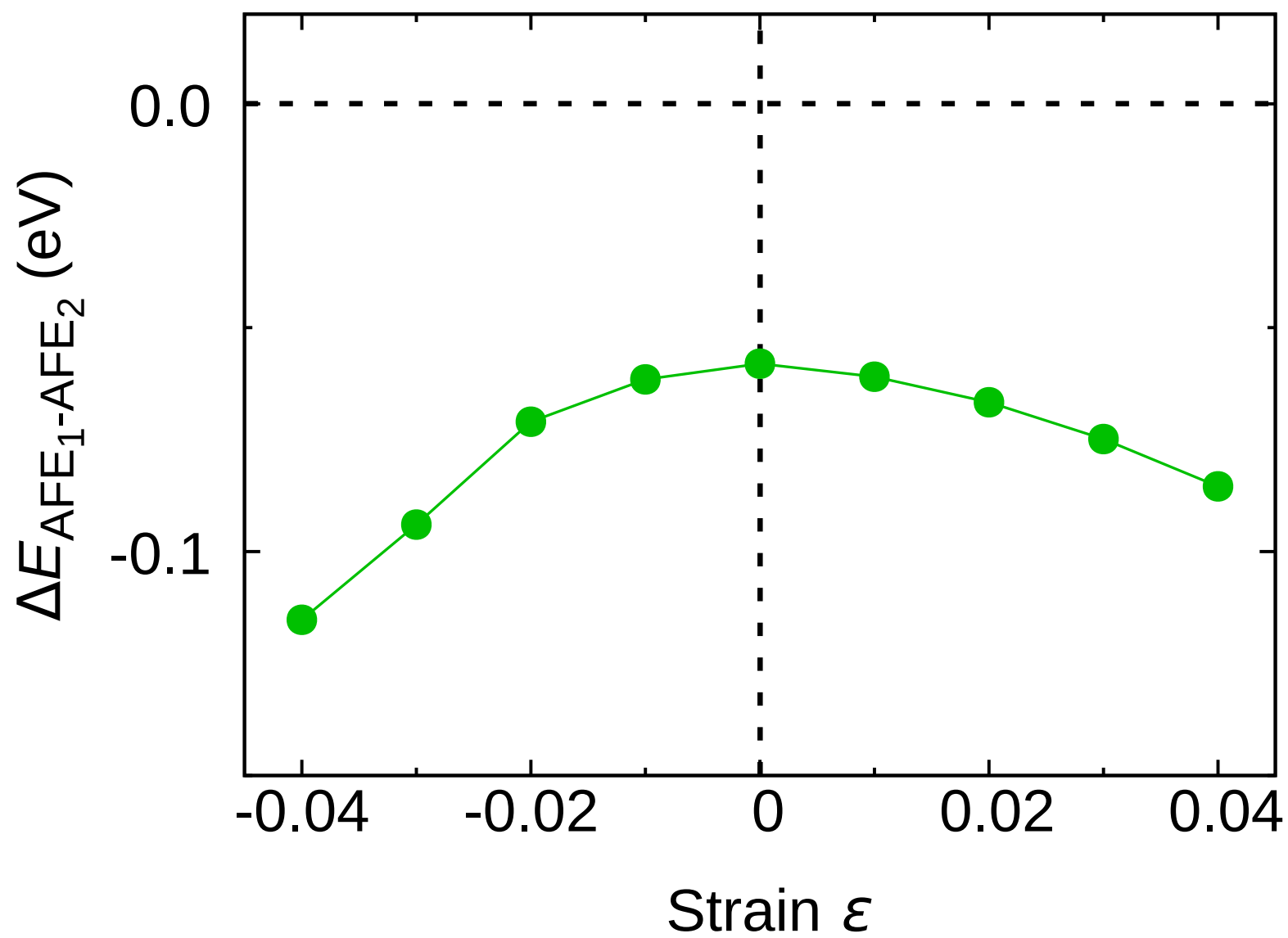

Figure S3: Strain dependence of energy difference between $\mathrm{AFE_1}$ and $\mathrm{AFE_2}$. The negative $\triangle E$ values indicate that $\mathrm{AFE_1}$ remains energetically favored over $\mathrm{AFE_2}$ throughout the considered strain range.

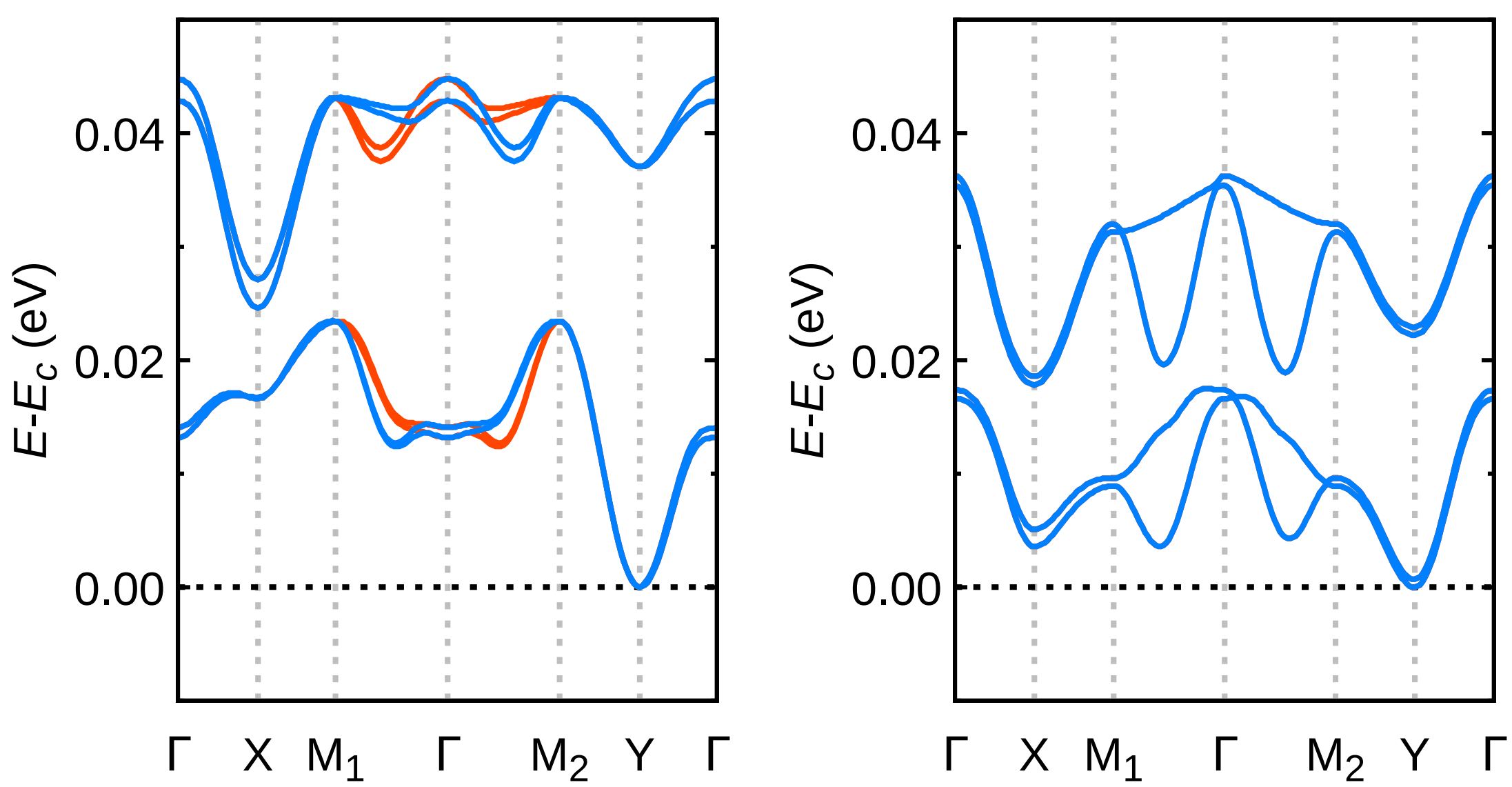

Figure S4: Spin-polarized band structures of the bulk-stacked exfoliated bilayer $\mathrm{AFE_1}$ and $\mathrm{AFE_2}$. To further assess whether these symmetry-distinct magnetic states survive beyond the monolayer limit, we also considered isolated bilayers exfoliated from the bulk crystal while preserving the original bulk stacking sequence. After full structural relaxation, the bulk-stacked $\mathrm{AFE_1}$ bilayer belongs to the layer group $pb2_1a$, in which the two opposite-spin sublattices are connected by the spin symmetry operations $[C_2 \parallel C_{2x}t]$ and $[C_2 \parallel M_y t]$, thereby retaining the characteristic altermagnetic spin splitting. In contrast, the bulk-stacked $\mathrm{AFE_2}$ bilayer belongs to the layer group $p2_12_12$, where the two opposite-spin sublattices are connected by $[C_2 \parallel C_{2z}t]$, which protects the spin-degenerate type-IV AFM state in the nonrelativistic limit. The spin-polarized band structures of the bulk-stacked bilayers further confirm these symmetry assignments. Therefore, exfoliation that preserves the bulk stacking sequence does not change the symmetry distinction between $\mathrm{AFE_1}$ and $\mathrm{AFE_2}$: the $\mathrm{AFE_1}$ bilayer retains the AM state, whereas the $\mathrm{AFE_2}$ bilayer retains the type-IV AFM state.
